# Phylogenetic and CRISPR/Cas9 Studies in Deciphering the Evolutionary Trajectory and Phenotypic Impacts of Rice *ERECTA* Genes

**DOI:** 10.3389/fpls.2018.00473

**Published:** 2018-04-10

**Authors:** Yanchun Zhang, Simin Li, Shijie Xue, Sihai Yang, Ju Huang, Long Wang

**Affiliations:** State Key Laboratory of Pharmaceutical Biotechnology, School of Life Sciences, Nanjing University, Nanjing, China

**Keywords:** *ERECTA* gene, phylogeny, homologs, rice population genetics, gene function, CRISPR/Cas9

## Abstract

The *ERECTA* family genes (*ERfs*) have been found to play diverse functions in *Arabidopsis*, including controlling cell proliferation and cell growth, regulating stomata patterning, and responding to various stresses. This wide range of functions has rendered them as a potential candidate for crop improvement. However, information on their functional roles, particularly their morphological impact, in crop genomes, such as rice, is limited. Here, through evolutionary prediction, we first depict the evolutionary trajectory of the *ER* family, and show that the *ER* family is actually highly conserved across different species, suggesting that most of their functions may also be observed in other plant species. We then take advantage of the CRISPR/Cas9 (clustered regularly interspaced short palindromic repeats–associated nuclease 9) system to assess their morphological impact on one of the most important crops, rice. Loss-of-function mutants of *OsER1* and *OsER2* display shortened plant stature and reduced panicle size, suggesting they possibly also functioned in regulating cell proliferation and cell growth in rice. In addition to functions similar to that in *Arabidopsis*, we also find clues that rice *ERfs* may play unique functional roles. The *OsER2* displayed more severe phenotypic changes than *OsER1*, indicating putative differentiation in their functions. The *OsERL* might be of essential in its function, and the proper function of all three rice *ER* genes might be dependent of their genetic background. Future investigations relating to these functions are key to exploiting *ERfs* in crop development.

## Introduction

The *ERECTA* (*ER*) gene has been extensively studied in *Arabidopsis thaliana* for its famous Landsberg *erecta* (L*er*) ecotype (Torii et al., 1996; van Zanten et al., 2009; Shpak, 2013). *Arabidopsis* plants with homozygous *er* alleles have very short inflorescence stems, siliques, and pedicels (Torii et al., 1996). The *ER* gene encodes a leucine-rich repeat receptor-like kinase (LRR-RLK) that has functional Ser/Thr kinase activity, which in turn plays a regulatory role in plant development. During *Arabidopsis* organogenesis, the *ER* gene, together with other RLK members, plays a critical role in controlling cell proliferation and cell growth (Shpak et al., 2003). The ER pathway could regulate shoot apical meristem (SAM) size and floral meristem identity in parallel to the class III HD-ZIP (homeodomain-leucine-zipper) and CLV (CLAVATA) pathways (Mandel et al., 2014, 2016). Natural allelic variations in the *Arabidopsis* *ER* locus have been associated with variations in petal shape, suggesting its function in determining petal shape (Abraham et al., 2013). In addition to these morphological functions, ER protein also functions as plant a physiological regulator. The *ER* gene was found to regulate transpiration efficiency in *Arabidopsis* (Masle et al., 2005), overexpression of ER significantly increases the thermotolerance in *Arabidopsis*, rice, and tomato (Qi et al., 2004; Shen et al., 2015). The *ER* gene has also been reported to be a contributor to shade-avoidance syndrome, possibly working in a background-dependent manner (Kasulin et al., 2013). Finally, being a member of the LRR family, which is best described in resistance (R) genes, ER also affects the resistance to various biotic and abiotic factors (Godiard et al., 2003; Shpak, 2013; Jordá et al., 2016; Takahashi et al., 2016).

Generally, the *ER* gene functions together with its family members. There are two paralogs of *ER* in *Arabidopsis*, namely, *ER-LIKE1* (*ERL1*) and *ER-LIKE2* (*ERL2*). Throughout the secondary growth of *Arabidopsis* hypocotyl, *ER* and *ERL1* redundantly prevent premature progression of sequential events (Ikematsu et al., 2017). Three *ERECTA* family genes (*ERfs*) could also interact synergistically in regulating stomatal patterning (Shpak et al., 2005) and mediating morphological alterations (Uchida et al., 2011). Therefore, the *ER* family acts cooperatively or redundantly in regulating almost every aspect of plant development (Shpak, 2013; Kosentka et al., 2017; Tameshige et al., 2017; Qu et al., 2017).

Due to its function in regulating plant development and thermotolerance, the *ER* family may be potentially utilized in agriculture. Rice (*Oryza sativa*) is the most important food resource in Asian countries, and there is currently a need to increase its yield to feed continuously growing human populations. It is thus essential to identify and characterize functional genes in rice that may assist in improving yield, quality, and resistance. There are several *ERfs* present in the rice genome (referred to as *OsERfs* hereafter). The overexpression of *OsER1* increases the heat tolerance of transgenic rice, whereas *OsER1* plants carrying the T-DNA null allele died quickly when grown at high temperatures (Shen et al., 2015), thereby suggesting that the *ER* family plays a critical functional role in rice, and has the potential to be used to breed thermotolerant crops.

However, current knowledge on *ERfs* has been mostly derived from *Arabidopsis.* Functional studies on *ERfs* are lagged behind in other crops, such as rice, particularly relating to morphological impacts, thus hindering their application to agricultural programs. The *OsER1* gene has only been investigated in terms of its role in thermotolerance (Shen et al., 2015), whereas its phenotypic influences remain unclear. Furthermore, studies on the functional role in response to stresses of *OsERL*, which also known as *OsSIK1*, are conflicting. Previous studies have shown that the overexpression of *OsERL* increases the antioxidant capacity of rice, indicating that it plays an important role in responding to salt and drought stress, possibly by suppressing stomatal development in rice leaves (Ouyang et al., 2010). However, a more recent study did not observe any difference in heat tolerance between *OsERL* mutants and the wild-type control (Shen et al., 2015), and thus *OsERL*’s regulatory role in stomatal development remains elusive. Whether these discrepancies among studies were due to different treatments, i.e., salt and drought stresses used by Ouyang et al. (2010) compared to heat stress in Shen et al. (2015), or other factors remain unclear. To our knowledge, no functional assessment of *OsER2* has been conducted to date, which is partly due to an incorrect gene structure as well as gene symbol given to *OsER2* in public rice databases (see section “Discussion” for details).

The poor understanding of rice *ERfs* is also attributable to the difficulty in obtaining desired mutants with specific genetic backgrounds. The CRISPR/Cas9 system has emerged as a revolutionary genome editing tool that allows targeted mutagenesis at base pair (bp) resolution. In this study, we investigated: (1) whether all of the *ERfs* in rice have similar functions as that in *Arabidopsis*; and (2) whether three *OsERfs* have unique features that differ from those in *Arabidopsis*. To answer these, we first predicted the putative functions of *OsERfs* based on evolutionary analyses, and then we knocked out three *OsERfs* in the rice genome via the CRISPR/Cas9 system to confirm their functional impact on plant phenotypic characteristics. Our results show that *ERfs* are highly conserved across the plant kingdom, hence some of their functions could be extrapolated from *Arabidopsis* to rice. However, different *OsERfs* also indicate addition functions other than that reported in *Arabidopsis*. We also reveal the putative influence of genetic background on *OsERf* function that has not been described in previous reports. These results pave the way for further utilization of *ERfs* in modern breeding programs.

## Materials and Methods

### Identification of *ERfs* and Construction of Phylogenetic Tree

Gene sequences of 56 selected plant species were collected from JGI Phytozome database v12.1 (Goodstein et al., 2012), NCBI GenBank, and individual genome project websites (Supplementary Table S1). The protein sequence of *Arabidopsis ER* gene was used as a seed to search for *ERfs* of all selected plants through NCBI tblastn (Altschul et al., 1990) with *E*-value cutoff set to 0.01. Only blast hits with identity ≥50% and coverage ≥50% were defined as *ERfs* to distinguish from other LRR-RLK members (Supplementary Table S2). The protein sequences of all identified *ERfs* were aligned by MUSCLE (Edgar, 2004) with default parameters. The aligned protein sequences were further used to guide the alignments of CDS. A phylogenetic tree was constructed using the Neighbor-Joining (NJ) method with 1,000 replicates of bootstrap test through MEGA 6 (Tamura et al., 2013). Each identified gene was classified as *ER* or *ERL-LIKE (ERL)* based on its similarity to *Arabidopsis ER* gene and was further confirmed by its position in the phylogenetic tree.

### Estimation of Genetic Parameters of *ERfs*

Sequence similarity and *Ka, Ks* values of *ER* genes and *ERL* genes across selected plant species were calculated using MEGA 6 (Supplementary Table S3). For within-population analysis, 247 high-coverage re-sequenced rice cultivars (Supplementary Table S4) were collected from the 3,000 rice genomes project (The 3000 Rice Genomes Project, 2014). Four groups were sampled, including *indica* (IND), *aus* (AUS), *temperate japonica* (TEJ), and *tropical japonica* (TRJ), the *aromatic* (ARO) group was not included due to too limited sample size. The cleaned reads were mapped to the Nipponbare reference genome Os-Nipponbare-Reference-IRGSP-1.0 (Kawahara et al., 2013) using BWA-MEM v0.7.10-r789 (Li, 2013) with default parameters. Variants were called using GATK HaplotypeCaller module (McKenna et al., 2010), and were filtered by removing variant calls with a quality <50. The population-level phylogenetic tree was constructed using variant loci with a minor allele frequency (MAF) ≥0.05. The average nucleotide diversities within different rice subspecies were calculated using public available PERL script “calc_vcf_diversity.pl” ^1^ as described in Wang et al. (2016).

Putative effects of variants in rice *ERfs* were predicted using SNPeffect 4.0 (De Baets et al., 2012) against MSU Rice Genome Annotation Project (RGAP) Release 7 database (Kawahara et al., 2013). The results for *OsER2* were further examined manually based on NCBI model. Tajima’s *D*-value was estimated using DnaSP v5 (Librado and Rozas, 2009).

### Generate of Mutant Plants of Rice *ERfs* by CRISPR/Cas9

Spacers were designed according to Shan et al. (2014) for three rice *ERfs*. The complementary oligonucleotides of each spacer were inserted into BsaI restriction site of plasmid of single-guide RNAs (sgRNAs). Then sgRNA were incorporated into the Cas9 vector, which contained Cas9 driven by the maize ubiquitin promoter with Gateway recombination method (Katzen, 2007). The vectors were then transformed into two rice cultivars *Oryza sativa* L. ssp. *indica* cultivar Kasalath and *Oryza sativa* L. ssp. *japonica* cultivar Wuyungeng24. Kasalath was a traditional variety with high tolerance to unsatisfactory growing conditions such as drought and phosphate deficiency (Sakai et al., 2014). It has been extensively used in rice functional characterization and the genome sequences were available (Sakai et al., 2014). Wuyungeng24 was chose as a representative cultivar of *japonica* rice for its good fertility.

### Verification of Mutagenesis

Genomic DNA of transgenic lines was extracted using protocols suggested by Monna et al. (2002). To identify mutations in regenerated plants, genomic DNA surrounding the targeted regions of sgRNAs was amplified by PCR and afterwards sequenced by Sanger et al. (1977) method. As *OsER1* and *OsER2* share a high similarity, both genes were verified in regenerated plants of either *OsER1* or *OsER2* to ensure no off-targeted editing. Plant lines with mutated alleles as well as WT were selected for phenotypic analyses.

## Results

### *ER* Family Emerged Early in Plants and Is Highly Conserved in Terms of Copy Number and Gene Structure Across Different Species

**FIGURE 1:**
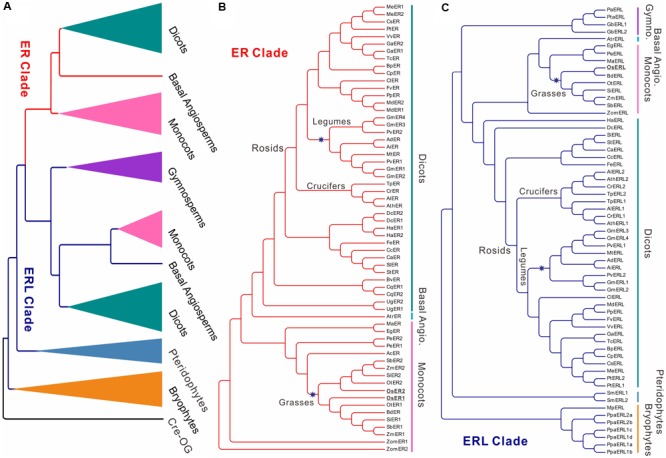
Cladogram of *ERECTA* family genes *(ERfs)* among 49 plant species. The cladogram was constructed using protein sequences of all identified *ERfs*. One LRR protein Cre17.g741150.t2.1 (Cre-OG) from *Chlamydomonas reinhardtii* was picked as an outgroup. Two major clades, *ERECTA* (*ER*) clade and *ER-LIKE* (*ERL*) clade were identified based on their relationship to *Arabidopsis* ER and ERL proteins. **(A)** Simplified cladogram of ER and ERL proteins across major divisions (e.g., kingdoms or sub-kingdoms). Each clade and group are indicated by different colors; **(B)** Cladogram of all ER proteins across 49 plant species; **(C)** Cladogram of all ERL proteins across 49 plant species. Major divisions are shown on the right side with the same color schemes as used in **(A)**; two whole-genome duplication (WGD) events specific to grasses and legumes are indicated by blue stars; the rice ER and ERL proteins were highlighted are underlined. The abbreviations for each species were given in **Table 1**. A full phylogenetic tree with bootstrap values was given in Supplementary Figure S1. Abbreviation: Gymno., Gymnosperms; Basal Angio., Basal Angiosperms.

The protein sequence of the *A. thaliana ER* gene was used as query sequence to search for its homologs in all of the collected genomes (see section “Materials and Methods” for details). No homologs with protein identity ≥50% were found in all of the seven algae (**Table 1**, BLAST in six species from Chlorophyta and one species *Klebsormidium flaccidum* from Charophyta). The most ancestral *ER* family with *ERL* members only was found in bryophytes, while the first *ER* gene was found in basal angiosperms (**Figure 1**, Supplementary Figure S1, and **Table 1**), suggesting that the *ER* family only appeared in early land plants (i.e., Embryophyta). All of the collected *ERfs* were clearly separated into two large clades, which we denoted as the *ER* clade and *ERL* clade based on their relationship to the *Arabidopsis ER* and *ERL* genes (**Figure 1A** and Supplementary Figure S1).

**Table 1:** Numbers of identified *ERECTAs* (*ERs)* and *ER-LIKEs (ERLs)* in 56 selected plant species.

Species (Common name)	Abbreviation	No. of *ERs*	No. of *ERLs*
**Chlorophyta**			
*Ostreococcus tauri*	–	0	0
*Ostreococcus lucimarinus*	–	0	0
*Micromonas pusilla*	–	0	0
*Volvox carteri*	–	0	0
*Chlamydomonas reinhardtii*	–	0	0
*Dunaliella salina*	–	0	0
**Bryophytes**			
*Klebsormidium flaccidum*	–	0	0
*Marchantia polymorpha*	Mp	0	1
*Physcomitrella patens*	Ppa	0	6
**Vascular plants**			
**Pteridophytes**			
*Selaginella moellendorffii*	Sm	0	2
**Gymnosperms**			
*Ginkgo biloba*	Gb	0	2
*Picea abies*	Pa	0	1
*Pinus taeda*	Pta	0	1
**Basal Angiosperms**			
*Amborella trichopoda*	Atr	1	1
**Monocots**			
*Zostera marina*	Zom	2	1
*Phalaenopsis equestris* (Orchid)	Pe	2	1
*Elaeis guineensis* (Oil palm)	Eg	1	1
*Ananas comosus* (Pineapple)	Ac	1	0
*Musa acuminata* (Banana)	Ma	1	1
*Oropetium thomaeum*	Ot	2	1
*Zea mays* (Maize)	Zm	2	1
*Sorghum bicolor*	Sb	2	1
*Setaria italica* (Foxtail Millet)	Si	2	1
*Brachypodium distachyon*	Bd	1	1
*Oryza sativa* (Rice)	Os	2	1
**Dicots**			
**Caryophyllales**			
*Beta vulgaris* (Sugar beet)	Bv	1	0
*Chenopodium quinoa*	Cq	2	0
**Asterids**			
*Fraxinus excelsior* (European ash)	Fe	1	1
*Utricularia gibba*	Ug	2	0
*Capsicum annuum* (Peppers)	Ca	1	1
*Solanum Lycopersicon* (Tomato)	Sl	1	1
*Solanum tuberosum* (Potato)	St	1	1
*Coffea canephora* (Coffee)	Cc	1	1
*Daucus carota* (Carrot)	Dc	2	1
*Helianthus annuus* (Sunflower)	Ha	2	1
*Vitis vinifera* (Grape)	Vv	1	1
**Rosids**			
**Fabids**			
*Arachis duranensis* (Wild Peanut)	Ad	1	1
*Arachis ipaensis* (Wild Peanut)	Ai	1	1
*Medicago truncatula*	Mt	1	1
*Glycine max* (Soybean)	Gm	4	4
*Phaseolus vulgaris* (Common bean)	Pv	2	2
*Malus domestica* (Apple)	Md	2	1
*Prunus persica* (Peach)	Ppe	1	1
*Fragaria vesca* (Strawberry)	Fv	1	1
*Betula pendula* (Silver birch)	Bp	1	1
*Citrullus lanatus* (Watermelon)	Cl	1	1
*Manihot esculenta* (Cassava)	Me	2	1
*Populus trichocarpa* (Poplar)	Ptr	1	2
**Malvids**			
*Citrus sinensis* (Orange)	Cs	1	1
*Theobroma cacao*	Tc	1	1
*Gossypium arboreum* (Cotton)	Ga	2	1
*Carica papaya* (Papaya)	Cp	1	1
*Arabidopsis thaliana*	Ath	1	2
*Arabidopsis lyrata*	Al	1	2
*Capsella rubella*	Cr	1	2
*Thellungiella parvula*	Tp	1	2

*No ERECTA family genes (ERfs) are found in Chlorophyta. The abbreviations are used in gene names and phylogenetic trees. Some major divisions are given in bold. Several common names are given in brackets.*

It is noteworthy that the *ERfs* of gymnosperms exclusively belong to the *ERL* clade (**Figure 1C**), suggesting that the *ER* clade only emerged from the basal angiosperms. The number of *ER* genes in each species remains steady across all of the subsequent species with only one or two copies, roughly corresponding to the whole-genome duplication (WGD) events that occurred in each of the species (**Figure 1B**). The soybean genome possesses the largest *ER* family with four *ER* genes and four *ERL* genes, which were possibly contributed by multiple WGD events (Schmutz et al., 2010). However, the first expansion of the *ER* family in the soybean genome most likely occurred prior to its divergence from common bean (also containing two copies of *ER* and *ERL*), but after their divergence from other legumes (only having one *ER* and *ERL*), thus not due to the legume-specific WGD (**Figures 1B,C** and **Table 1**).

We have identified three *ERfs* in the rice genome, including two *ER* genes, *OsER1* (*LOC_Os06g10230/Os06g0203800*) and *OsER2* (*LOC_Os02g53720/Os02g0777400*), and one *ERL* gene *OsERL* (*LOC_Os06g03970/Os06g0130100*) (**Figure 1** and Supplementary Figure S1). Unlike legumes, *OsER1* and *OsER2* were possibly produced due to the grass-specific WGD (Paterson et al., 2009; Zhang et al., 2012), as most other grass species also contain two copies of the *ER* genes (**Figure 1B** and **Table 1**). Compared to the *ER* genes, the *ERL* genes seemed to have an independent evolutionary path, as suggested by the asynchronous copy number variations compared to the *ER* genes in most species (**Figure 1C** and **Table 1**). Besides a possible different evolutionary history of *ERs* and *ERLs*, the whole *ER* family seemed to be highly conserved with no rapid loss or expansion found both across and within species.

In addition to conservation in gene number, the gene structure of *ERfs* also remained unchanged among most species (Supplementary Table S2). All of the *ER* genes surveyed here have 22∼29 introns, whereas approximately 90% of the *ERL* genes contain 25∼27 introns (Supplementary Table S2).

### Functional Constraints of *ERfs* as Revealed by Signatures of Negative Selection Between and Within Species

Molecular signatures of selection have been proven to be a powerful tool in generating functional inferences (Nielsen, 2005). Therefore, we investigated whether the *ER* family has undergone any selection forces due to its pleiotropic functions. We found a relatively high similarity of ER proteins (78.0% in average) and *ERL* genes (76.3% in average) across all of the angiosperms, despite a long evolutionary history (Supplementary Table S3). We then compared the number of non-synonymous changes per non-synonymous site (*Ka*) and the corresponding number of synonymous changes per synonymous site (*Ks*) values across all of the selected species for each of the *ER* and *ERL* genes. Both *ER* genes as well as the *ERL* gene displayed 10-fold lower *Ka* values compared to their *Ks* values (Supplementary Table S3), indicating strong negative selections. This agrees with the observed conserved function role of the *ER* family.

A similar signature of negative selection was also detected within rice populations. Among 247 rice cultivars analyzed (Supplementary Table S4), only 12, 4, and 7 single nucleotide polymorphisms (SNPs) with MAF ≥0.05 were detected within the coding sequences (CDS) of *OsER1, OsER2*, and *OsERL*, respectively (Supplementary Table S5). *OsER1* and *OsER2* both harbor only two non-synonymous changes, whereas *OsERL* contains four polymorphism sites that could alter the encoded amino acids. No insertions/deletions (indels) were found with a MAF ≥0.05 within the coding regions of those genes.

All three genes have limited diversities and small *Ka/Ks* ratios (Supplementary Table S5), suggesting putative negative selections as observed across species, although this signature was not significant in Tajima’s *D*-test (Supplementary Table S5). However, one thing that seemed to be clear is that the allele distributions were subjected to the fine structure of the rice population (Supplementary Figure S2A). The population structure of Asian cultivated rice has been extensively reshaped by strong artificial selections, which could be divided into two major subspecies, i.e., *indica* and *japonica*, and further subdivided in to five groups: *indica* (IND), *aus* (AUS), *aromatic* (ARO), *temperate japonica* (TEJ), and *tropical japonica* (TRJ) (Garris et al., 2005). The selected 247 rice accessions contain four major groups, consisting 120 IND and 27 AUS, which belong to *indica* subspecies, and 52 TEJ and 48 TRJ, which belong to *japonica* subspecies (Supplementary Table S4).

Four major haplotype groups (Hap1∼4) of *OsER1* were identified based on the linkages of 12 SNP sites (Supplementary Figure S2B). Hap1 showed the highest frequency (∼68%) and was detected in all four of the rice groups (Supplementary Figure S2 and Supplementary Table S6), thus this haplotype could be viewed as the ancestral haplotype that was present before the sub-differentiation of cultivated rice. The Hap2 differed from Hap1 only on one SNP, but was mainly found in TEJ and TRJ from *japonica* subspecies (Supplementary Figure S2). The two other haplotypes, Hap3 and Hap4, which both distinct from Hap2, could only be found in *indica* groups (Supplementary Figure S2). There were as few as four SNPs between Hap3 and Hap4 (Supplementary Figure S2). Therefore, those three haplotypes stand for latterly generated polymorphisms during the sub-speciation of *indica* and *japonica* rice, and their distances from the ancestral haplotype Hap1 (Supplementary Figure S2) coincided with an earlier origin of *japonica* rice, which is predated to *indica* rice (Huang et al., 2012; Yuan et al., 2017).

Unlike *OsER1, OsER2* lacks an ancestral haplotype shared by all four of the groups (Supplementary Figure S2), indicating two genes possibly diverged after their derivation from the WGD event in grasses. The subsequent evolution of *OsER2* generated three major haplotypes (Hap1 and Hap2 with a frequency over 30%, and Hap3 with a frequency of only 3%) with very limited variants (Supplementary Figure S2B and Supplementary Table S6), and it also followed the differentiation of two subspecies with Hap1, corresponding to *indica*, and Hap2 and Hap3, corresponding to *japonica* rice (Supplementary Figure S2A).

In contrast to *OsER1* and *OsER2, OsERL* showed a moderate admixture of different groups (Supplementary Figure S2A), again suggesting an independent evolutionary path of *OsERL* compared to the *OsER* genes. Nonetheless, genetic polymorphisms are limited among all of the major haplotypes of *OsERL* (Supplementary Figure S2B).

### *OsERfs* Have Similar Expression Patterns as *Arabidopsis ER* Genes

The expression level of the *Arabidopsis ER* genes is directly linked to their regulatory functions (Shpak, 2013); therefore, we further investigated the expression patterns of *OsERfs*. Three *OsERfs*, especially *OsER1* and *OsERL*, were found to share a similar expression level across different organs (Supplementary Figures S3A,C). Three *OsERfs* showed the highest expression in inflorescences, e.g., spikelet, panicles, and stamens, etc. A high to medium expression level was observed in leaves, shoots, and stems. The roots showed the lowest expression levels. This expression pattern was largely the same as that observed in *Arabidopsis* (Torii et al., 1996; Supplementary Figure S3C).

The expression levels among different developmental stages were more variable in rice, but the general trend did not differed much from that of *Arabidopsis* (Supplementary Figures S3B,D). The highest expression level was found in the early stage of vegetative growth, and decreased to an intermediate level at the later phase of the reproductive stage.

In addition to these observed similarities, unlike *OsER1* and *OsERL, OsER2* also displayed distinct expression patterns, as compared to *Arabidopsis*. In sperm cells, *OsER2* showed extremely high expression levels, whereas *OsER1, OsERL*, and *Arabidopsis ERfs* were not expressed (Supplementary Figures S3A,C). *OsER2* was also upregulated during heading stage, whereas the other genes were not (Supplementary Figure S3B). These distinct expression patterns indicate that *OsER2* possibly plays a distinct role compared to the other rice *ER* genes.

### Generation of *OsERf* Mutants Using the CRISPR/Cas9 System

**FIGURE 2:**
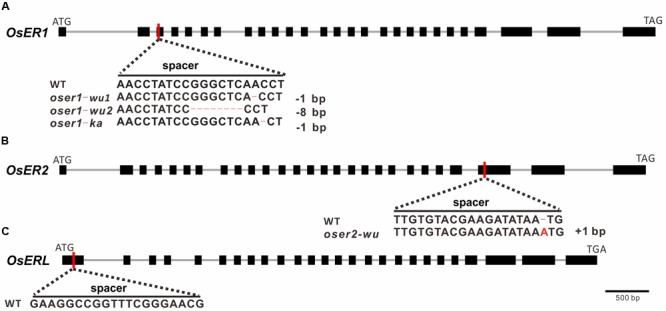
Targeted sites, designed spacers, and obtained mutant types for **(A)** *OsER1*, **(B)** *OsER2*, and **(C)** *OsERL* gene. Red vertical lines represent the targeted sites of designed spacers within the coding sequence. Bases edited by the CRISPR/Cas9 system are highlighted with colored bases or dash lines. No mutants were obtained for *OsERL* after two independent transformation trails.

Three sgRNAs (spacers) were designed to target each *ERf* for precise mutagenesis. To minimize the possibility of off-targeted editing, the spacer sequence was designed with no other similar matches (BLAST hits allow no more than five mismatches) except for the target regions. The spacer for *OsER1* and *OsER2* was designed to target the front-end of their 3rd and 25th exons, respectively (**Figures 2A,B**), whereas the spacer for *OsERL* was designed at the 1st exon (**Figure 2C**). Two rice varieties, *O. sativa* L. ssp. *indica* cultivar Kasalath (KA) and *O. sativa* L. ssp. *japonica* cultivar Wuyungeng24 (WU), were selected as the recipients for their transformation efficiency. Through *Agrobacterium*-mediated transformation, the CRISPR/Cas9 construct was introduced into rice embryogenic calli, resulting in 13 regenerated transgenic plants (T0 generation) of KA background and 47 regenerated transgenic plants of WU background. PCR amplification followed by Sanger sequencing was used to confirm whether the vectors were successfully transformed into the transgenic plants (Supplementary Table S7). Since *OsER1* and *OsER2* share a high similarity, when testing the targeted region in regenerated plants of *OsER1*, we also verified its best-matched region in *OsER2*. No mutation was found within the best-matched region of *OsER2*, suggesting no off-target effect. This was also true for the regenerated plants of *OsER2*.

Of the 13 KA-T0 plants, there were five regenerated plants for *OsER1* and eight for *OsERL*, but no successfully regenerated plants were obtained for *OsER2* after three independent transformation trials. Two of the five *OsER1-KA* plants were successfully mutated at the designed sites (a 40% mutation rate), whereas no mutations were found in the targeted regions of all eight of the *OsERL-KA* plants compared to the wild type (WT) line. Of the 47 WU-T0 plants, the mutagenesis efficiency was generally higher, with all seven of the *OsER1-WU* plants and all 20 of the *OsER2-WU* plants exhibiting successful gene knockout (a 100% mutation rate). Unfortunately, still no mutations were found in the designed regions of all 20 of the *OsERL-WU* plants. Finally, we failed to generate null mutants for *OsERL* after another round of independent transformation trials. This raised the possibility that the *OsERL* functioned as an essential gene that would be lethal when totally lost. In sum, we have obtained nine *oser1* mutant plants, with two with a KA background and seven with a WU background, and 20 *oser2* mutant plants, all with the WU background.

DNA analysis revealed two mutant types for *OsER1-WU* plants, one mutant type for *OsER1-KA* and *OsER2-WU* plants (**Figures 2A,B**). Both *oser1-wu1* and *oser1-ka* mutant type contain -1 bp (at position +227 bp and +228 bp of CDS, respectively) frameshift mutations, whereas the *oser1-wu2* type has an 8 bp (at position +220 bp) deletion, and *oser2-wu* harbors a 1 bp (at position +1,940 bp) insertion. All of those frameshift mutations would introduce premature stop codons at protein position 88, 92, 88, and 651 in the *oser1-wu1, oser1-wu2, oser1-ka*, and *oser2-wu* mutants, respectively.

### *OsER* Mutants Have Shortened Statures, Reduced Panicle Size, and Seed Setting Rate Compared to the WT

**FIGURE 3:**
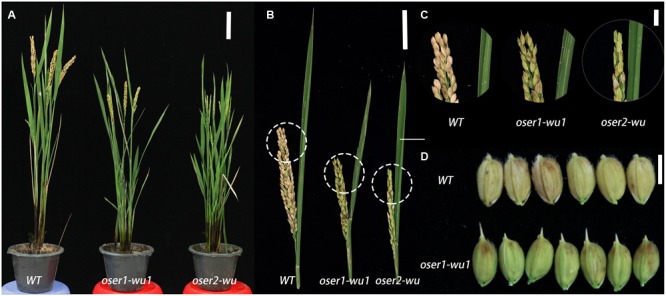
Phenotypic analyses of mutant plants for *OsER1* and *OsER2*. **(A)** Phenotypes of mature plants of *oser1-wu1* and *oser2-wu* mutants. **(B)** Changes in panicle size in the *oser1-wu1* and *oser2-wu* mutants compared to the WT; **(C)** Enlarged view of the panicles of each plant as circled in **(B)**; **(D)** Morphology of seeds of *oser1-wu1* compared to the WT, no detectable phenotypic changes observed in seeds of *oser2-wu*. Bars = 10 cm **(A)**, 5 cm **(B)**, 1 cm **(C)**, and 5 mm **(D)**.

To assess the phenotypic impact of the *oser* mutants, the T1 generation of three *oser1-wu1*, six *oser1-wu2*, six *oser1-ka*, and 10 *oser2-wu* plants were obtained through selfing of the T0 plants. During the maturing stage, no obvious phenotypic changes were observed in the *oser1-ka* mutants. In contrast, all of the *oser* mutants with the WU background, i.e., *oser1-wu1, oser1-wu2*, and *oser2-wu*, showed significantly shortened plant stature as well as reduced panicle size compared to the WT (**Figures 3A,B** and Supplementary Figures S4A,B). Since no obvious difference was observed between *oser1-wu1* and *oser1-wu2* mutants, we only focused on *oser1-wu1* mutants hereafter to avoid repetition. The *oser2-wu* mutants exhibited a more severe dwarf phenotype compared to *oser1-wu1* (**Figures 3A,B** and Supplementary Figures S4A,B). Not only the plant stature but also the panicle size of *oser2-wu* mutants were significantly shorter than *oser1-wu1* (two-tailed Students’ *t*-test, *P* = 0.00163 for plant height and *P* = 2.55 ×10^-5^ for panicle size).

The *oser1-wu1* mutant also have slightly elongated awns of seeds compared to WT (**Figure 3C**), while the similar short awns were not observed in *oser2-wu*. On the contrary, there was no significant difference of seed setting rate between *oser1-wu1* and WT, but a significant impaired seed setting rate was found in *oser2-wu* (**Figure 3D** and Supplementary Figure S4C). In conclusion, both *OsER* genes played a role in control plant height and panicle size of rice WU plants, while *OsER2* might be also involved in regulation of reproductive process.

## Discussion

### The Evolutionary Trajectory of the *ER* Family

The *ER* family has been shown to play pleotropic functional roles during various development stages of *Arabidopsis* (Shpak, 2013); however, whether those functions also occur in other plant species remains unclear. We hypothesize that, owing to the important roles of the *ER* family, many of its functions are preserved in most plant species. The present study provides four pieces of evidence that support this hypothesis.

First, a gene’s function usually could constraint the evolution route of itself, and in turn, an overview of its phylogeny could provide clues on its function (Eisen, 1998). In this study, we investigated the evolutionary trajectory of the *ER* family among diverse plant species. A previous study had surveyed 12 plant species, from which the *ERfs* were found to be present in a very early stage during land-plant evolution (Villagarcia et al., 2012). Here, we expanded the phylogenetic analysis to include a total of 56 plant species (**Figure 1**, Supplementary Figure S1, **Table 1**, and Supplementary Table S1), encompassing almost every node of the Viridiplantae, thereby offering a picture with unprecedented high resolution for the evolutionary history of the *ER* family. We find the *ERfs*, especially *ERs*, were rarely lost after their emergence, indicating a functional constraint on those genes.

Second, all of the *ERfs* have an intact gene structure, which contains multiple introns. In *Arabidopsis*, the presence of multiple introns in the *ER* gene was supposed to be essential for its expression, and those introns increase *ER* expression in an additive manner (Karve et al., 2011). Therefore, the structure of multiple introns or splicing patterns could be a key character of *ERfs*. The intact gene structure is also found in rice genome, suggesting a preserved *ER* and *ERL* gene structure across different plant species, coinciding with its importance in regulating its expression.

However, we did find several species that contain fewer introns within *ERLs* (Supplementary Table S2), which could be due to two possible reasons. One is that the *ERLs*, unlike *ERs*, might face less constraint in their expressions, as suggested by the independent evolution path. Another is that some of these annotated *ERLs* in public databases were incomplete due to imperfect gene predictions. Here, we prefer the later answer. Even in the well-annotated rice genome, we found that the two most often used database, i.e., MSU and RAP-DB, both predicted a truncated version of *OsER2* (Supplementary Figure S5A). Only the NCBI model captures the correct structure of *OsER2*, which is also supported by the RNA-seq data (Supplementary Figure S5B). Although RAP-DB assigned the “OsER2” symbol to gene *Os06g0130100*, phylogenetic analysis reveals that this gene actually belongs to the *ERL* clade, and the bona fide *OsER2* gene is the complete version of *LOC_Os02g53720/Os02g0777400* as given by the NCBI model (*LOC4330905*). Therefore, we suggest that caution be exercised in using *OsER2* and *OsERL* from public databases.

The third piece of evidence came from the within-species diversities of rice population. There were only limited polymorphisms among *OsERfs*, and most diversities were established before rice sub-speciation as implied in *OsER1*. The sub-speciation process leads to the fixation of differentiated polymorphisms in the *OsER1* and *OsER2*, but not in *OsERL*, which is in line with their independent evolutionary trajectory. All of the three genes displayed limited variations owing to their functional constraints.

Finally, besides the conservation witnessed at the variants level, an unchanged expression pattern again hints that their functions could be largely preserved in rice compared to *Arabidopsis*. The expression pattern in *Arabidopsis* reflects their functional roles in regulating cell proliferation and expansion in vegetative and reproductive organs (Shpak, 2013). Therefore, the observed similar expression patterns between rice and *Arabidopsis* suggest that most of these functions may also be applicable to rice. In conclusion, all the above evidences suggest that the *ER* family is one of the most conserved subfamilies in LRR-RLK genes (Liu et al., 2017).

From all above evidences, we could now delineate a picture of how *ERfs* evolved across different species. Given the *ER* family’s functional role in stomatal patterning, is it possible that the emergence of the *ER* family coincided with the emergence of stomata in land plants? However, a contrasting fact is that one *ERL* gene was found in the liverwort *Marchantia polymorpha* (**Figure 1C** and **Table 1**), which actually lacks stomata (Bowman et al., 2017). Further evidence was found in the genome of seagrass *Zostera marina* (**Figure 1** and **Table 1**). *Z. marina* is a marine angiosperm that has lost its stomata as well as all the genes involved in stomatal differentiation (Olsen et al., 2016), whereas the *ER* family was preserved (**Figure 1** and **Table 1**). These findings suggest that the early *ER* family may have functioned in other aspects and was only later involved in the regulation networks of stomatal patterning.

The *ERLs* emerged prior to *ERs*, and the first *ERL* appeared with the emergence of the whole LRR-RLK family, which was also absent in algal species (Liu et al., 2017). As mentioned above, the early *ERLs* may have not differed much from other LRR-RLK members and possibly did not possess those functions specific to *ERfs*. Since the first ER was only found in *Amborella*, suggesting the true origin of *ERs* could be basal angiosperms. Besides a different time of origin, *ERs* and *ERLs* also evolved via two independent routes. The expansion of *ERs* followed well with the WGD events that occurred in different clades, whereas this pattern was rarely observed in the *ERLs*. At the level of rice populations, the evolution of the *ERs* consisted of sub-speciation process, whereas the *ERLs* did not. The independent evolutionary path of the *ERs* and *ERLs* may explain the observed distinct functions between *ERs* and *ERLs* in some species (Shen et al., 2015).

Combined with the cross-species analyses, we could also delineate the whole history of the three *OsERfs. OsER1*, and *OsER2* originated from a duplication of their common ancestor during the grass-specific WGD, and were both involved in the sub-speciation process of rice population. In contrast, *OsERL* might has lost one copy after WGD, and its subsequent evolution was less subjected to the rice sub-speciation progress.

### The Phenotypic Impacts of Rice *ER* Genes

The *Arabidopsis ER* gene was first recognized by its functional roles in contributing to a shortened inflorescence stems, siliques, and pedicels (Torii et al., 1996). A similar phenomenon was observed here in rice with a reduced panicle size in *oser1-wu* and *oser2-wu* mutants. In *Arabidopsis*, three *ERfs* interacted synergistically in controlling plant organ size, where *ERL1* and *ERL2* functioned redundantly, and the loss of all *ERfs* leads to severed dwarfism in plants (Shpak et al., 2004). In rice, the two *ERs* also functioned with a certain level of redundancy, while the loss of either gene could cause a shortened stature in rice plants. Whether this change in plant height reflected a similar function of rice *ERs* in cell proliferation and cell growth as *Arabidopsis* (Shpak et al., 2003) still needs more evidences on cellular level, though. The similarity in the phenotypic impacts of the *ER* genes in controlling plant architecture between *Arabidopsis* and rice again emphasizes the conserved functional characters of the *ER* genes, and suggests that many of their functions as well as mechanisms could be readily to migrate to other plant species, as also indicated by their regulatory roles in thermotolerance among various species (Qi et al., 2004; Shen et al., 2015).

However, some caveats are still necessary when migrating those functions. First, the functions of *ERs* seemed to be dependent on their genetic background in rice. Distinct from the significantly changed morphological traits in *oser1-wu* and *oser2-wu* mutants, the *oser1-ka* mutation conferred no detectable phenotype. Sequence analysis revealed only one SNP in the penultimate exon between the *OsER1*-KA allele (belonging to the Hap1 in Supplementary Figure S2) and the *OsER1*-*WU* allele (belonging to the Hap2 in Supplementary Figure S2). Another two small indels (≤3 bp) and three SNPs were found between two cultivars within the intron and promoter regions (defined as 3 kb apart from the start codon) of *OsER1*, respectively. Similarly, the *OsER2* differed in only four SNPs between KA (belonging to the Hap1 in Supplementary Figure S2) and WU (belonging to the Hap2 in Supplementary Figure S2), of which two were non-synonymous changes. These limited differences of *OsER1* and *OsER2* might could not fully explain the distinct phenotypes between *oser1-ka* and *oser1-wu*. Since the two selected cultivars belong to different subspecies of rice, this suggests the putative functional diversification of *OsERs* between *indica* and *japonica* rice, which is also depicted in their differentiated evolutionary paths as discussed earlier. It is also curious to note that all of the previous investigation on *OsER1* and *OsERL* were conducted exclusively in *japonica* rice (Ouyang et al., 2010; Shen et al., 2015). Whether this was simply due to a greater convenience of transformation in *japonica* rice as compared to *indica* rice, or was indeed subjected to its genetic backgrounds awaits further confirmation.

Second, although different *oser* mutants shared many phenotypical changes, the *oser2* mutants always have more severed impacts than *oser1*, indicating unique functional characters for each of the *OsER* genes besides of their redundant functions. This was more pronounced in their influences on seed setting rate, which was only observed in the mutants of *OsER2*. The functional role of *OsER2* in rice reproduction is in line with its uniquely elevated expression level in sperm cells and during the heading stage (Supplementary Figures S3A,B). Therefore, although *OsER1* and *OsER2* still share many characters in common, there could have been substantial functional divergence between two genes during grass evolution.

Finally, although we failed to assess the functions of *OsERL*, some evidence suggests different roles for *OsERL* compared to *OsERs* and *Arabidopsis ERfs*. One piece of evidence was reported previously when dissecting OsER1’s function in thermotolerance, which showed that *OsERL* is not involved in thermos-response in rice using T-DNA insertional mutant (Shen et al., 2015). Although Shen et al. (2015) also obtained a T-DNA mutant for *OsERL* and confirmed that its expression level was abolished, the T-DNA was actually inserted into the 24th exon, which was quite near its terminus. The mutants used by Ouyang et al. (2010) were also near the end of the gene, with a *Tos17* insertion in the 19th exon and the 23rd intron of *OsERL*, but they observed that the mutants performed differently under salt and drought treatments. Our *OsERL* spacer was designed at the very beginning of the gene (**Figure 2**), which did not successfully generate mutant plants. Therefore, we propose that the basic function of *OsERL* is essential which could not bear a completely loss, but part of their function, such as stress-tolerance, relies on how many exons were preserved, similar to that observed in *Arabidopsis* (Karve et al., 2011). Much like the functional roles of *OsERL*, evolutionary analysis shows that the *ERLs* underwent an independent evolutionary trajectory compared to *ERs*, and rice only contains one copy of *ERL* whereas *Arabidopsis* has two, thereby resulting in different functional constraints.

Notwithstanding most of these suggestions need to be further tested, the presence of several unique functional characters in rice *ERfs* compared to *Arabidopsis* seems to be true. Future exploiting of *ERfs* into crop development should be aware of these differences.

## Author Contributions

LW and JH designed the project. YZ, LW, SL, and SX performed the experiments and analyzed the data. LW wrote the paper. YZ prepared the figures and tables. JH and SY revised the paper. All authors read and approved the final article.

## Conflict of Interest Statement

The authors declare that the research was conducted in the absence of any commercial or financial relationships that could be construed as a potential conflict of interest.

## Footnotes

1 https://github.com/wl13/BioScripts/blob/master/calc_vcf_diversity.pl
