# Supplementary material for: Phylogenetic and CRISPR/Cas9 Studies in Deciphering the Evolutionary Trajectory and Phenotypic Impacts of Rice ERECTA Genes
Source: Front Plant Sci. 2018 Apr 10;9:473. doi: 10.3389/fpls.2018.00473 (PMC5902711; doi:10.3389/fpls.2018.00473)
Supplement: Supplementary file 1 [file Table_1.DOCX]

**Table S1. List of all 56 selected plant species and data source used in this study.**

| **Species** | **Release** | **Source** |
| --- | --- | --- |
| *Ostreococcus tauri* | NCBI (GCA_000214015.2) | <https://www.ncbi.nlm.nih.gov/genome/> |
| *Ostreococcus lucimarinus* | Phytozome (V12) | https://genome.jgi.doe.gov/ |
| *Micromonas pusilla* | Phytozome (V12) | https://genome.jgi.doe.gov/ |
| *Volvox carteri* | Phytozome (V12) | https://genome.jgi.doe.gov/ |
| *Chlamydomonas reinhardtii* | Phytozome (V12) | https://genome.jgi.doe.gov/ |
| *Dunaliella salina* | Phytozome (V12) | https://genome.jgi.doe.gov/ |
| *Klebsormidium flaccidum* | Algae genome project (v1.1) | [http://www.plantmorphogenesis.bio.titech.ac.jp](http://www.plantmorphogenesis.bio.titech.ac.jp/~algae_genome_project/klebsormidium/) |
| *Marchantia polymorpha* | Phytozome (V12) | https://genome.jgi.doe.gov/ |
| *Physcomitrella patens* | Phytozome (V12) | https://genome.jgi.doe.gov/ |
| *Selaginella moellendorffii* | Phytozome (V12) | https://genome.jgi.doe.gov/ |
| *Ginkgo biloba* | Treegenes (v1.0) | http://treegenesdb.org/FTP/Genomes/ |
| *Picea abies* | Treegenes (v1.0b) | http://treegenesdb.org/FTP/Genomes/ |
| *Pinus taeda* | Treegenes (v2.01) | http://treegenesdb.org/FTP/Genomes/ |
| *Amborella trichopoda* | Phytozome (V12) | https://genome.jgi.doe.gov/ |
| *Zostera marina* | Phytozome (V12) | https://genome.jgi.doe.gov/ |
| *Phalaenopsis equestris* | Coge (v1.0, id25065) | <https://genomevolution.org/CoGe/> |
| *Elaeis guineensis* | NCBI (GCA_000442705.1) | https://www.ncbi.nlm.nih.gov/genome/ |
| *Ananas comosus* | Phytozome (V12) | https://genome.jgi.doe.gov/ |
| *Musa accuminata* | Banana-genome-hub (v2) | http://banana-genome-hub.southgreen.fr/ |
| *Oropetium thomaeum* | Phytozome (V12) | https://genome.jgi.doe.gov/ |
| *Zea mays* | Phytozome (V12) | https://genome.jgi.doe.gov/ |
| *Sorghum bicolor* | Phytozome (V12) | https://genome.jgi.doe.gov/ |
| *Setaria italica* | Phytozome (V12) | https://genome.jgi.doe.gov/ |
| *Brachypodium distachyon* | Phytozome (V12) | https://genome.jgi.doe.gov/ |
| *Oryza sativa* | NCBI (GCA_001433935.1) | https://www.ncbi.nlm.nih.gov/genome/ |
| *Beta vulgaris* | RefBeet (v1.1) | http://bvseq.molgen.mpg.de/Genome/ |
| *Chenopodium quinoa* | ChenopodiumDB (v1) | http://www.cbrc.kaust.edu.sa/chenopodiumdb/ |
| *Fraxinus excelsior* | Treegenes (v0.5) | <http://treegenesdb.org/FTP/Genomes/> |
| *Utricularia gibba* | Coge (v4.1, id25895) | <https://genomevolution.org/CoGe/> |
| *Capsicum annuum* | ZYAAS (v2.0) | <http://peppersequence.genomics.cn/> |
| *Solanum Lycopersicon* | Phytozome (V12) | https://genome.jgi.doe.gov/ |
| *Solanum tuberosum* | NCBI (GCA_000226075.1) | https://www.ncbi.nlm.nih.gov/genome/ |
| *Coffea canephora* | CGH (v1.0) | http://coffee-genome.org/download |
| *Daucus carota* | Phytozome (V12) | https://genome.jgi.doe.gov/ |
| *Helianthus annuus* | HanXRQ-SUNRISE (v1.2) | https://www.heliagene.org/ |
| *Vitis vinifera* | Phytozome (V12) | https://genome.jgi.doe.gov/ |
| *Arachis duranensis* | PeanutBase (V14167) | https://peanutbase.org/data/public/ |
| *Arachis ipaensis* | PeanutBase (K30076.gnm1.ann1.J37m) | https://peanutbase.org/data/public/ |
| *Medicago truncatula* | Phytozome (V12) | https://genome.jgi.doe.gov/ |
| *Glycine max* | Phytozome (V12) | https://genome.jgi.doe.gov/ |
| *Phaseolus vulgaris* | Phytozome (V12) | https://genome.jgi.doe.gov/ |
| *Malus domestica* | Phytozome (V12) | https://genome.jgi.doe.gov/ |
| *Prunus persica* | Phytozome (V12) | https://genome.jgi.doe.gov/ |
| *Fragaria vesca* | Phytozome (V12) | https://genome.jgi.doe.gov/ |
| *Betula pendula* | Coge (1.4c Pseudochromosomes, id35080) | <https://genomevolution.org/CoGe/> |
| *Citrullus lanatus* | Curcurbit Genomics Database (97103 v1) | ftp://cucurbitgenomics.org/pub/ |
| *Manihot esculenta* | Phytozome (V12) | https://genome.jgi.doe.gov/ |
| *Populus trichocarpa* | Phytozome (V12) | https://genome.jgi.doe.gov/ |
| *Citrus sinensis* | Phytozome (V12) | https://genome.jgi.doe.gov/ |
| *Theobroma cacao* | Phytozome (V12) | https://genome.jgi.doe.gov/ |
| *Gossypium arboreum* | BGI (v2.0) | https://www.cottongen.org/species |
| *Carica papaya* | Phytozome (V12) | https://genome.jgi.doe.gov/ |
| *Arabidopsis thaliana* | Phytozome (V12) | https://genome.jgi.doe.gov/ |
| *Arabidopsis lyrata* | Phytozome (V12) | https://genome.jgi.doe.gov/ |
| *Capsella rubella* | Phytozome (V12) | https://genome.jgi.doe.gov/ |
| *Thellungiella parvula* | Thellungiella (v2.0) | http://thellungiella.org/data/ |
