# Supplementary material for: Phylogenetic and CRISPR/Cas9 Studies in Deciphering the Evolutionary Trajectory and Phenotypic Impacts of Rice ERECTA Genes
Source: Front Plant Sci. 2018 Apr 10;9:473. doi: 10.3389/fpls.2018.00473 (PMC5902711; doi:10.3389/fpls.2018.00473)
Supplement: Supplementary file 2 [file Table_2.DOCX]

**Table S2. CDS length, exon number and sequence similarity of all 122 identified *ERfs* across 49 plant species.** The sequence similarities are given as identity to *Arabidopsis EREACTA* (*AthER*) gene here. The annotation id refers to the original id in the source databases. For rice *OsER2*, the NCBI model was used instead of the truncated version in MSU and RAP-DB. Three *ERfs* in *Oropetium thomaeum* were also supposed to be truncated and re-annotated locally using Fgenesh (Salamov and Solovyev, 2000). The three re-annotated sequences have been deposited in NCBI genbank under accession OtER1MG882071-3.

| **Gene Symbol** | **Annotation ID** | **CDS length** | **No. of exons** | **Identity to *AthER* (%)** |
| --- | --- | --- | --- | --- |
| ***ERs*** |  |  |  |  |
| *AcER* | Aco023274.1 | 3,267 | 29 | 76.2 |
| *AdER* | Aradu.F19Z7 | 2,919 | 25 | 77.6 |
| *AiER* | Araip.B0I4X | 2,976 | 25 | 76.3 |
| *AlER* | AL4G18950.t1 | 2,931 | 26 | 98.7 |
| *AthER* | AT2G26330.1 | 2,931 | 26 | 100.0 |
| *AtrER* | evm_27.model.AmTr_v1.0_scaffold00024.267 | 2,988 | 26 | 75.2 |
| *BdER* | Bradi1g46450.1 | 2,937 | 26 | 73.5 |
| *BpER* | Bpev01.c0064.g0001.m0001 | 2,967 | 26 | 83.0 |
| *BvER* | Bv6_134910_mkyn.t1 | 2,979 | 26 | 79.3 |
| *CaER* | Capana08g000433 | 2,946 | 26 | 79.6 |
| *CcER* | Cc08_g01090 | 2,979 | 26 | 82.2 |
| *ClER* | Cla007455 | 2,976 | 26 | 78.8 |
| *CpER* | evm.model.supercontig_126.17 | 2,523 | 25 | 84.7 |
| *CqER1* | AUR62009075RA | 2,982 | 26 | 78.7 |
| *CqER2* | AUR62020592RA | 2,982 | 26 | 78.6 |
| *CrER* | Carubv10025215m | 2,931 | 26 | 96.1 |
| *CsER* | orange1.1g002349m | 2,802 | 24 | 78.0 |
| *DcER1* | DCAR_004377 | 2,394 | 22 | 67.1 |
| *DcER2* | DCAR_003718 | 2,619 | 25 | 75.3 |
| *EgER* | XP_010917192.1 | 2,961 | 26 | 76.8 |
| *FeER* | FRAEX38873_v2_000106980.2 | 2,898 | 26 | 79.5 |
| *FvER* | mrna15491.1v1.0hybrid | 3,168 | 28 | 77.4 |
| *GaER1* | Cotton_A_05648_BGIA2_v1.0 | 2,952 | 26 | 81.6 |
| *GaER2* | Cotton_A_39203_BGIA2_v1.0 | 2,694 | 23 | 57.4 |
| *GmER1* | Glyma.17G226100.1 | 2,943 | 26 | 81.2 |
| *GmER2* | Glyma.14G098600.1 | 2,943 | 26 | 81.0 |
| *GmER3* | Glyma.04G056200.1 | 2,958 | 26 | 79.7 |
| *GmER4* | Glyma.06G056400.1 | 2,955 | 26 | 79.7 |
| *HaER1* | HanXRQChr09g0261811 | 2,961 | 25 | 79.8 |
| *HaER2* | HanXRQChr05g0135931 | 2,961 | 25 | 78.6 |
| *MaER* | Ma07_t02250.1 | 2,943 | 26 | 77.1 |
| *MdER1* | MDP0000223968 | 2,976 | 28 | 78.0 |
| *MdER2* | MDP0000144320 | 3,009 | 26 | 79.2 |
| *MeER1* | Manes.16G086000.1 | 2,970 | 26 | 83.1 |
| *MeER2* | Manes.03G053600.1 | 2,952 | 26 | 80.3 |
| *MtER* | Medtr1g015530.1 | 2,958 | 26 | 77.3 |
| *OsER1* | Os06t020380001 | 2,937 | 26 | 74.0 |
| *OsER2* | LOC4330905 | 2,961 | 26 | 72.7 |
| *OtER1* | Oropetium_20150105_17208A | 2,871 | 25 | 71.2 |
| *OtER2* | Oropetium_20150105_04392A | 2,469 | 19 | 52.6 |
| *PeER1* | PEQU_11285 | 2,868 | 25 | 73.3 |
| *PeER2* | PEQU_03869 | 2,961 | 26 | 73.9 |
| *PpER* | Prupe.1G403600.2 | 2,976 | 26 | 82.5 |
| *PtER* | Potri.006G220100.1 | 2,943 | 26 | 81.3 |
| *PvER1* | Phvul.001G038400.1 | 2,940 | 26 | 81.7 |
| *PvER2* | Phvul.009G082466.1 | 1,719 | 23 | 81.6 |
| *SbER1* | Sobic.010G077000.3 | 2,946 | 26 | 74.3 |
| *SbER2* | Sobic.004G317200.1 | 3,018 | 26 | 70.9 |
| *SiER1* | Seita.4G086700.1 | 2,973 | 26 | 74.0 |
| *SiER2* | Seita.1G338900.1 | 2,901 | 25 | 69.4 |
| *SlER* | Solyc08g061560.2.1 | 2,973 | 26 | 80.0 |
| *StER* | PGSC0003DMT400048435 | 2,973 | 26 | 81.1 |
| *TcER* | Thecc1EG037393t1 | 2,952 | 26 | 82.5 |
| *TpER* | Tp4g06960 | 2,934 | 26 | 94.4 |
| *UgER1* | Scf00040.g4689.t1 | 2,967 | 22 | 68.4 |
| *UgER2* | Scf00375.g16514.t1 | 2,745 | 23 | 70.8 |
| *VvER* | GSVIVT01035476001 | 2,961 | 26 | 83.9 |
| *ZmER1* | GRMZM5G809695_T02 | 2,985 | 26 | 73.4 |
| *ZmER2* | GRMZM2G463904_T03 | 3,000 | 26 | 69.9 |
| *ZomER1* | Zosma87g00130.1 | 2,955 | 26 | 71.2 |
| *ZomER2* | Zosma292g00090.1 | 2,937 | 23 | 64.0 |
| **Average** | | 2,903 | 25 | 77.7 |
| ***ERLs*** |  |  |  |  |
| *AdERL* | Aradu.F623P | 2,988 | 26 | 63.2 |
| *AiERL* | Araip.F3F2F | 2,988 | 26 | 62.7 |
| *AlERL1* | AL6G17400.t1 | 2,901 | 26 | 64.6 |
| *AlERL2* | AL8G39050.t1 | 2,901 | 26 | 63.8 |
| *AthERL1* | AT5G07180.1 | 2,904 | 26 | 62.1 |
| *AthERL2* | AT5G62230.1 | 2,901 | 26 | 63.2 |
| *AtrERL* | evm_27.model.AmTr_v1.0_scaffold00069.214 | 2,934 | 26 | 66.3 |
| *BdERL* | Bradi1g49950.1 | 2,949 | 25 | 65.0 |
| *BpERL* | Bpev01.c1334.g0012.m0001 | 2,976 | 26 | 62.9 |
| *CaERL* | Capana03g002487 | 2,646 | 26 | 66.0 |
| *CcERL* | Cc02_g03790 | 2,847 | 25 | 64.3 |
| *ClERL* | Cla009454 | 3,024 | 26 | 64.9 |
| *CpERL* | evm.model.supercontig_3.259 | 2,961 | 26 | 63.7 |
| *CrERL1* | Carubv10002808m | 2,904 | 27 | 64.7 |
| *CrERL2* | Carubv10025821m | 2,913 | 26 | 62.2 |
| *CsERL* | orange1.1g002278m | 2,832 | 25 | 64.2 |
| *DcERL* | DCAR_018130 | 2,928 | 26 | 63.6 |
| *EgERL* | XP_010940921.1 | 2,967 | 26 | 65.1 |
| *FeERL* | FRAEX38873_v2_000379710.2 | 2,928 | 26 | 63.5 |
| *FvERL* | mrna13404.1v1.0hybrid | 2,973 | 26 | 63.5 |
| *GaERL* | Cotton_A_07558_BGIA2_v1.0 | 3,024 | 26 | 62.8 |
| *GbERL1* | Gb_26881 | 3,054 | 26 | 69.4 |
| *GbERL2* | Gb_33675 | 3,006 | 26 | 67.4 |
| *GmERL1* | Glyma.09G152400.1 | 2,955 | 26 | 62.1 |
| *GmERL2* | Glyma.16G203300.2 | 2,982 | 26 | 62.9 |
| *GmERL3* | Glyma.10G242300.1 | 2,970 | 26 | 63.2 |
| *GmERL4* | Glyma.20G151800.5 | 2,970 | 26 | 62.6 |
| *HaERL* | HanXRQChr15g0486121 | 2,916 | 26 | 63.0 |
| *MaERL* | Ma04_t26230.1 | 2,991 | 26 | 65.1 |
| *MdERL* | MDP0000159463 | 3,324 | 28 | 60.5 |
| *MeERL* | Manes.06G138900.1 | 3,063 | 26 | 63.8 |
| *MpERL* | Mapoly0070s0019.1 | 2,949 | 0 | 50.7 |
| *MtERL* | Medtr1g102500.1 | 2,982 | 26 | 62.1 |
| *OsERL* | Os06t013010001 | 3,000 | 25 | 63.1 |
| *OtERL* | Oropetium_20150105_16982A | 2,868 | 23 | 54.5 |
| *PaERL* | MA_4022g0010_PaERL | 1,641 | 13 | 57.7 |
| *PeERL* | PEQU_06599 | 1,764 | 10 | 58.4 |
| *PpaERL1a* | Pp3c19_15110V3.7 | 2,904 | 25 | 51.4 |
| *PpaERL1b* | Pp3c22_10630V3.2 | 2,937 | 25 | 48.8 |
| *PpaERL1c* | Pp3c18_10870V3.2 | 2,979 | 26 | 49.7 |
| *PpaERL1d* | Pp3c21_9500V3.1 | 2,976 | 26 | 50.9 |
| *PpaERL2a* | Pp3c2_22410V3.1 | 3,057 | 27 | 50.4 |
| *PpaERL2b* | Pp3c1_17360V3.4 | 2,880 | 27 | 49.3 |
| *PpERL* | Prupe.2G283600.1 | 2,961 | 26 | 62.9 |
| *PtaERL* | PITA_31519_PtaERL | 2,493 | 1 | 71.3 |
| *PtERL1* | Potri.015G132200.1 | 2,940 | 26 | 63.1 |
| *PtERL2* | Potri.012G130400.2 | 2,949 | 26 | 64.1 |
| *PvERL1* | Phvul.004G126100.1 | 2,970 | 26 | 62.6 |
| *PvERL2* | Phvul.007G063200.1 | 2,970 | 26 | 62.9 |
| *SbERL* | Sobic.010G020600.1 | 2,937 | 25 | 65.1 |
| *SiERL* | Seita.4G019700.1 | 2,937 | 25 | 65.1 |
| *SlERL* | Solyc03g007050.2.1 | 2,928 | 26 | 62.4 |
| *SmERL1* | 102522 | 2,835 | 24 | 62.0 |
| *SmERL2* | 114051 | 2,931 | 27 | 59.9 |
| *StERL* | XP_015167000.1 | 2,928 | 26 | 63.3 |
| *TcERL* | Thecc1EG015008t1 | 2,976 | 26 | 63.7 |
| *TpERL1* | Tp6g35720 | 2,883 | 26 | 61.4 |
| *TpERL2* | Tp2g25430 | 2,892 | 26 | 63.0 |
| *VvERL* | GSVIVT01018490001 | 2,793 | 26 | 66.6 |
| *ZmERL* | GRMZM2G082855_T01 | 2,958 | 25 | 64.2 |
| *ZomERL* | Zosma85g01030.1 | 3,045 | 26 | 60.0 |
| **Average** | | 2,900 | 25 | 62.0 |

**References**

Salamov, A. A., and Solovyev, V. V. (2000). Ab initio Gene Finding in Drosophila Genomic DNA. *Genome Res.* 10, 516–522. doi:10.1101/gr.10.4.516.
