# Supplementary material for: Phylogenetic and CRISPR/Cas9 Studies in Deciphering the Evolutionary Trajectory and Phenotypic Impacts of Rice ERECTA Genes
Source: Front Plant Sci. 2018 Apr 10;9:473. doi: 10.3389/fpls.2018.00473 (PMC5902711; doi:10.3389/fpls.2018.00473)
Supplement: Supplementary file 3 [file Table_3.DOCX]

**Table S3. Average protein similarities and *Ka*, *Ks* values within CDS regions of *ERs* and *ERLs* across all angiosperms.** The protein similarity was defined as “1- divergence (Dxy)”. The Dxy was estimated using MEGA 6 (Tamura et al., 2013) as average of pairwise p-distances. *Ka*, *Ks* values were calculated from aligned CDS, and the final value was averaged across all pairwise comparisons.

| Group | Protein similarity (%) | *Ka* | *Ks* | *Ka/Ks* |
| --- | --- | --- | --- | --- |
| *ERs* | 78.0 | 0.12 | 1.88 | 0.074 |
| *ERLs* | 76.3 | 0.14 | 2.09 | 0.079 |

**References**

Tamura, K., Stecher, G., Peterson, D., Filipski, A., and Kumar, S. (2013). MEGA6: Molecular Evolutionary Genetics Analysis Version 6.0. *Mol. Biol. Evol.* 30, 2725–2729. doi:10.1093/molbev/mst197.
