# Supplementary material for: Phylogenetic and CRISPR/Cas9 Studies in Deciphering the Evolutionary Trajectory and Phenotypic Impacts of Rice ERECTA Genes
Source: Front Plant Sci. 2018 Apr 10;9:473. doi: 10.3389/fpls.2018.00473 (PMC5902711; doi:10.3389/fpls.2018.00473)
Supplement: Supplementary file 4 [file Table_4.DOCX]

**Table S4. List of 247 selected rice cultivars from the 3,000 rice genomes project.**

| DNA UNIQUE ID |  | DNA UNIQUE ID |  | DNA UNIQUE ID |  | DNA UNIQUE ID |
| --- | --- | --- | --- | --- | --- | --- |
| **AUS** |  | **IND** |  | **IND** |  | **TEJ** |
| CX227 |  | IRIS_313-11644 |  | IRIS_313-11812 |  | IRIS_313-11651 |
| IRIS_313-10603 |  | IRIS_313-11645 |  | IRIS_313-11813 |  | IRIS_313-11652 |
| IRIS_313-10605 |  | IRIS_313-11646 |  | IRIS_313-11814 |  | IRIS_313-11661 |
| IRIS_313-10852 |  | IRIS_313-11648 |  | IRIS_313-11815 |  | IRIS_313-11725 |
| IRIS_313-10861 |  | IRIS_313-11656 |  | IRIS_313-11817 |  | IRIS_313-11800 |
| IRIS_313-10871 |  | IRIS_313-11657 |  | IRIS_313-11819 |  | IRIS_313-12060 |
| IRIS_313-10892 |  | IRIS_313-11664 |  | IRIS_313-11820 |  | IRIS_313-12061 |
| IRIS_313-11027 |  | IRIS_313-11665 |  | IRIS_313-11821 |  | IRIS_313-8125 |
| IRIS_313-11037 |  | IRIS_313-11668 |  | IRIS_313-11822 |  | IRIS_313-8151 |
| IRIS_313-11047 |  | IRIS_313-11669 |  | IRIS_313-11823 |  | IRIS_313-8502 |
| IRIS_313-11048 |  | IRIS_313-11671 |  | IRIS_313-12058 |  | IRIS_313-8755 |
| IRIS_313-11050 |  | IRIS_313-11674 |  | IRIS_313-8305 |  | IRIS_313-9379 |
| IRIS_313-11051 |  | IRIS_313-11681 |  | IRIS_313-8312 |  | IRIS_313-9701 |
| IRIS_313-11052 |  | IRIS_313-11683 |  | IRIS_313-8391 |  | **TRJ** |
| IRIS_313-11053 |  | IRIS_313-11684 |  | IRIS_313-8493 |  | B018 |
| IRIS_313-11056 |  | IRIS_313-11685 |  | IRIS_313-8925 |  | B190 |
| IRIS_313-11057 |  | IRIS_313-11686 |  | IRIS_313-8948 |  | CX106 |
| IRIS_313-11058 |  | IRIS_313-11687 |  | IRIS_313-9020 |  | CX111 |
| IRIS_313-11064 |  | IRIS_313-11692 |  | IRIS_313-9023 |  | CX151 |
| IRIS_313-11112 |  | IRIS_313-11700 |  | IRIS_313-9066 |  | CX220 |
| IRIS_313-11191 |  | IRIS_313-11704 |  | IRIS_313-9148 |  | CX243 |
| IRIS_313-11265 |  | IRIS_313-11705 |  | IRIS_313-9294 |  | CX262 |
| IRIS_313-11277 |  | IRIS_313-11707 |  | **TEJ** |  | CX269 |
| IRIS_313-11298 |  | IRIS_313-11708 |  | B001 |  | CX352 |
| IRIS_313-11737 |  | IRIS_313-11709 |  | B004 |  | CX355 |
| IRIS_313-11809 |  | IRIS_313-11710 |  | B014 |  | CX367 |
| IRIS_313-8864 |  | IRIS_313-11711 |  | B016 |  | IRIS_313-10541 |
| **IND** |  | IRIS_313-11716 |  | B017 |  | IRIS_313-10577 |
| B208 |  | IRIS_313-11717 |  | B045 |  | IRIS_313-10578 |
| B214 |  | IRIS_313-11719 |  | B046 |  | IRIS_313-10582 |
| B253 |  | IRIS_313-11720 |  | B066 |  | IRIS_313-10793 |
| B264 |  | IRIS_313-11721 |  | B160 |  | IRIS_313-10798 |
| B265 |  | IRIS_313-11722 |  | B162 |  | IRIS_313-10870 |
| IRIS_313-10171 |  | IRIS_313-11723 |  | B167 |  | IRIS_313-10872 |
| IRIS_313-10177 |  | IRIS_313-11727 |  | B182 |  | IRIS_313-10918 |
| IRIS_313-10609 |  | IRIS_313-11728 |  | B183 |  | IRIS_313-11044 |
| IRIS_313-10858 |  | IRIS_313-11730 |  | B204 |  | IRIS_313-11045 |
| IRIS_313-10863 |  | IRIS_313-11731 |  | B212 |  | IRIS_313-11046 |
| IRIS_313-10975 |  | IRIS_313-11738 |  | B225 |  | IRIS_313-11094 |
| IRIS_313-10986 |  | IRIS_313-11740 |  | B226 |  | IRIS_313-11102 |
| IRIS_313-11039 |  | IRIS_313-11744 |  | B235 |  | IRIS_313-11103 |
| IRIS_313-11083 |  | IRIS_313-11745 |  | B236 |  | IRIS_313-11104 |
| IRIS_313-11085 |  | IRIS_313-11746 |  | B250 |  | IRIS_313-11238 |
| IRIS_313-11089 |  | IRIS_313-11747 |  | B269 |  | IRIS_313-11659 |
| IRIS_313-11097 |  | IRIS_313-11748 |  | CX138 |  | IRIS_313-11673 |
| IRIS_313-11113 |  | IRIS_313-11750 |  | CX140 |  | IRIS_313-11736 |
| IRIS_313-11234 |  | IRIS_313-11752 |  | CX165 |  | IRIS_313-11739 |
| IRIS_313-11239 |  | IRIS_313-11760 |  | CX282 |  | IRIS_313-11755 |
| IRIS_313-11241 |  | IRIS_313-11761 |  | CX307 |  | IRIS_313-11759 |
| IRIS_313-11242 |  | IRIS_313-11762 |  | CX317 |  | IRIS_313-11790 |
| IRIS_313-11244 |  | IRIS_313-11786 |  | CX344 |  | IRIS_313-11929 |
| IRIS_313-11245 |  | IRIS_313-11789 |  | CX350 |  | IRIS_313-11994 |
| IRIS_313-11247 |  | IRIS_313-11794 |  | CX356 |  | IRIS_313-12129 |
| IRIS_313-11260 |  | IRIS_313-11795 |  | CX380 |  | IRIS_313-12164 |
| IRIS_313-11263 |  | IRIS_313-11796 |  | CX383 |  | IRIS_313-12228 |
| IRIS_313-11266 |  | IRIS_313-11797 |  | CX389 |  | IRIS_313-7883 |
| IRIS_313-11267 |  | IRIS_313-11798 |  | CX397 |  | IRIS_313-7902 |
| IRIS_313-11273 |  | IRIS_313-11801 |  | CX534 |  | IRIS_313-7914 |
| IRIS_313-11279 |  | IRIS_313-11802 |  | IRIS_313-10097 |  | IRIS_313-8010 |
| IRIS_313-11453 |  | IRIS_313-11804 |  | IRIS_313-10563 |  | IRIS_313-8173 |
| IRIS_313-11635 |  | IRIS_313-11805 |  | IRIS_313-10642 |  | IRIS_313-8356 |
| IRIS_313-11642 |  | IRIS_313-11810 |  | IRIS_313-11202 |  | IRIS_313-9470 |
| IRIS_313-11643 |  | IRIS_313-11811 |  |  |  |  |
