# Supplementary material for: Phylogenetic and CRISPR/Cas9 Studies in Deciphering the Evolutionary Trajectory and Phenotypic Impacts of Rice ERECTA Genes
Source: Front Plant Sci. 2018 Apr 10;9:473. doi: 10.3389/fpls.2018.00473 (PMC5902711; doi:10.3389/fpls.2018.00473)
Supplement: Supplementary file 5 [file Table_5.DOCX]

**Table S5. Genetic parameters of rice *ERfs* at population-level.**

| **Gene** | **π (Gene)** | **π (CDS)** | **Syn^a^** | **Non^b^** | ***Ka*** | ***Ks*** | ***Ka/Ks*** | **Tajima’s *D*** | **P-value^c^** |
| --- | --- | --- | --- | --- | --- | --- | --- | --- | --- |
| *OsER1* | 0.00165 | 0.00180 | 10 | 2 | 0.00024 | 0.0031 | 0.05 | -1.07 | P > 0.10 |
| *OsER2* | 0.00153 | 0.00126 | 2 | 2 | 0.00043 | 0.0014 | 0.31 | 0.11 | P > 0.10 |
| *OsERL* | 0.00199 | 0.00086 | 3 | 4 | 0.00043 | 0.0014 | 0.22 | -0.27 | P > 0.10 |

^a^ Number of synonymous changes; ^b^ Number of non-synonymous changes; ^c^ P-values were given for Tajima’s *D* test by DnaSP (Librado and Rozas, 2009).

**References**

Librado, P., and Rozas, J. (2009). DnaSP v5: a software for comprehensive analysis of DNA polymorphism data. *Bioinformatics* 25, 1451–1452. doi:10.1093/bioinformatics/btp187.
