# Supplementary material for: Phylogenetic and CRISPR/Cas9 Studies in Deciphering the Evolutionary Trajectory and Phenotypic Impacts of Rice ERECTA Genes
Source: Front Plant Sci. 2018 Apr 10;9:473. doi: 10.3389/fpls.2018.00473 (PMC5902711; doi:10.3389/fpls.2018.00473)
Supplement: Supplementary file 6 [file Table_6.DOCX]

**Table S6. Frequencies of major haplotype groups of *ERfs* within rice population.** Each haplotype group was defined in Figure S2.

|  | Haplotype Group | Frequency |
| --- | --- | --- |
| *OsER1* | Hap1 | 0.68 |
|  | Hap2 | 0.16 |
|  | Hap3 | 0.09 |
|  | Hap4 | 0.04 |
| *OsER2* | Hap1 | 0.59 |
|  | Hap2 | 0.03 |
|  | Hap3 | 0.38 |
| *OsERL* | Hap1 | 0.36 |
|  | Hap2 | 0.32 |
|  | Hap3 | 0.11 |
|  | Hap4 | 0.11 |
|  | Hap5 | 0.03 |
|  | Hap6 | 0.06 |
