# Supplementary material for: Phylogenetic and CRISPR/Cas9 Studies in Deciphering the Evolutionary Trajectory and Phenotypic Impacts of Rice ERECTA Genes
Source: Front Plant Sci. 2018 Apr 10;9:473. doi: 10.3389/fpls.2018.00473 (PMC5902711; doi:10.3389/fpls.2018.00473)
Supplement: Supplementary file 7 [file Table_7.DOCX]

**Table S7. Oligonucleotides used for construction of CRISPR/Cas9 vectors and mutation verification.** Primes were designed using OLIGO 7 software (Rychlik, 2007, 7). BsaI restriction sites are lowercased.

| Primer name | Sequence (5’-3’) |
| --- | --- |
| **Vector construction** | |
| *OsER1*-spacer-F | ggcaAAACCTATCCGGGCTCAACCT |
| *OsER1*-spacer-R | aaacAGGTTGAGCCCGGATAGGTT |
| *OsER2*-spacer-F | ggcaTTGTGTACGAAGATATAATG |
| *OsER2*-spacer-R | aaacCATTATATCTTCGTACACAA |
| *OsERL*-spacer-F | ggcaGAAGGCCGGTTTCGGGAACG |
| *OsERL*-spacer-R | aaacCGTTCCCGAAACCGGCCTTC |
| **Mutation verification** | |
| *OsER1*-F | GACGCTGCTGGAGATCAAG |
| *OsER1*-R | CGATACTGAGAACGGAATGTC |
| *OsER2*-F | AGGTCCCAGTCCATTGATT |
| *OsER2*-R | ACAGGCAACGGCTATACAA |
| *OsERL*-F | CCAGGGTCGATGCACTA |
| *OsERL*-R | GGGAGGCAAGAATAGAACA |

**References**

Rychlik, W. (2007). “OLIGO 7 Primer Analysis Software,” in *PCR Primer Design* Methods in Molecular Biology^TM^. (Humana Press), 35–59. doi:10.1007/978-1-59745-528-2_2.
