# Supplementary material for: Phylogenetic and CRISPR/Cas9 Studies in Deciphering the Evolutionary Trajectory and Phenotypic Impacts of Rice ERECTA Genes
Source: Front Plant Sci. 2018 Apr 10;9:473. doi: 10.3389/fpls.2018.00473 (PMC5902711; doi:10.3389/fpls.2018.00473)

Figure S1. Phylogenetic tree of *ERfs* among 49 plant species. The clades as well as divisions are colored the same as Figure 1. The rice ER and ERL proteins are underlined.

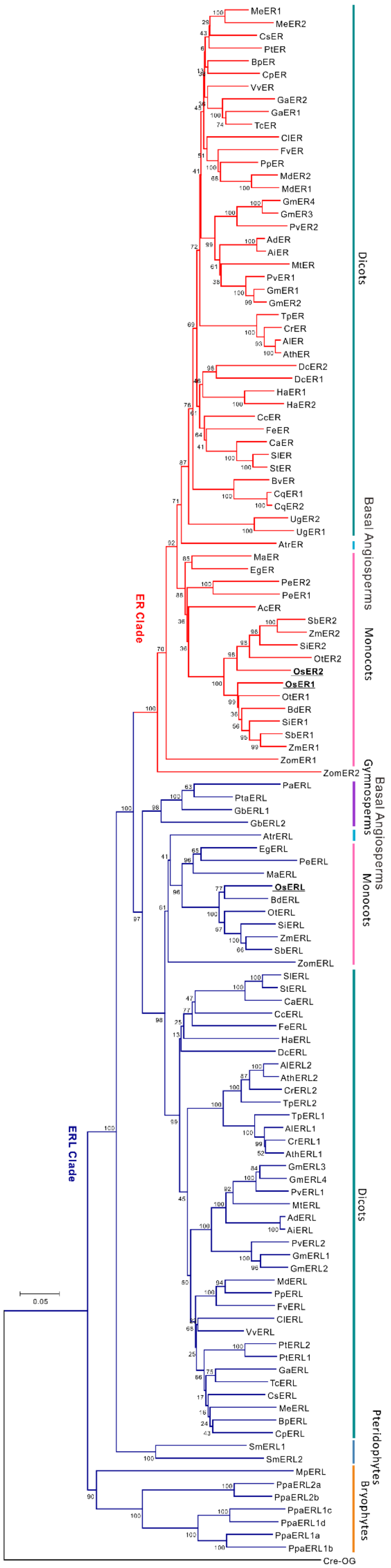

Supplement: Supplementary file 8 [file Image_1.PDF]
