# Supplementary material for: Phylogenetic and CRISPR/Cas9 Studies in Deciphering the Evolutionary Trajectory and Phenotypic Impacts of Rice ERECTA Genes
Source: Front Plant Sci. 2018 Apr 10;9:473. doi: 10.3389/fpls.2018.00473 (PMC5902711; doi:10.3389/fpls.2018.00473)
Supplement: Supplementary file 10 [file Image_3.PDF]

Figure S3. Expression patterns of *ERfs* in rice and *Arabidopsis*. The expression data was retrieved from Genevestigator database (Hruz et al., 2008). **(A)** Expression of rice *ERfs* among different tissues; **(B)** Expression of rice *ERfs* across different developmental stages; **(C)** Expression of *Arabidopsis ERfs* among different tissues; **(D)** Expression of *Arabidopsis ERfs* across different developmental stages.

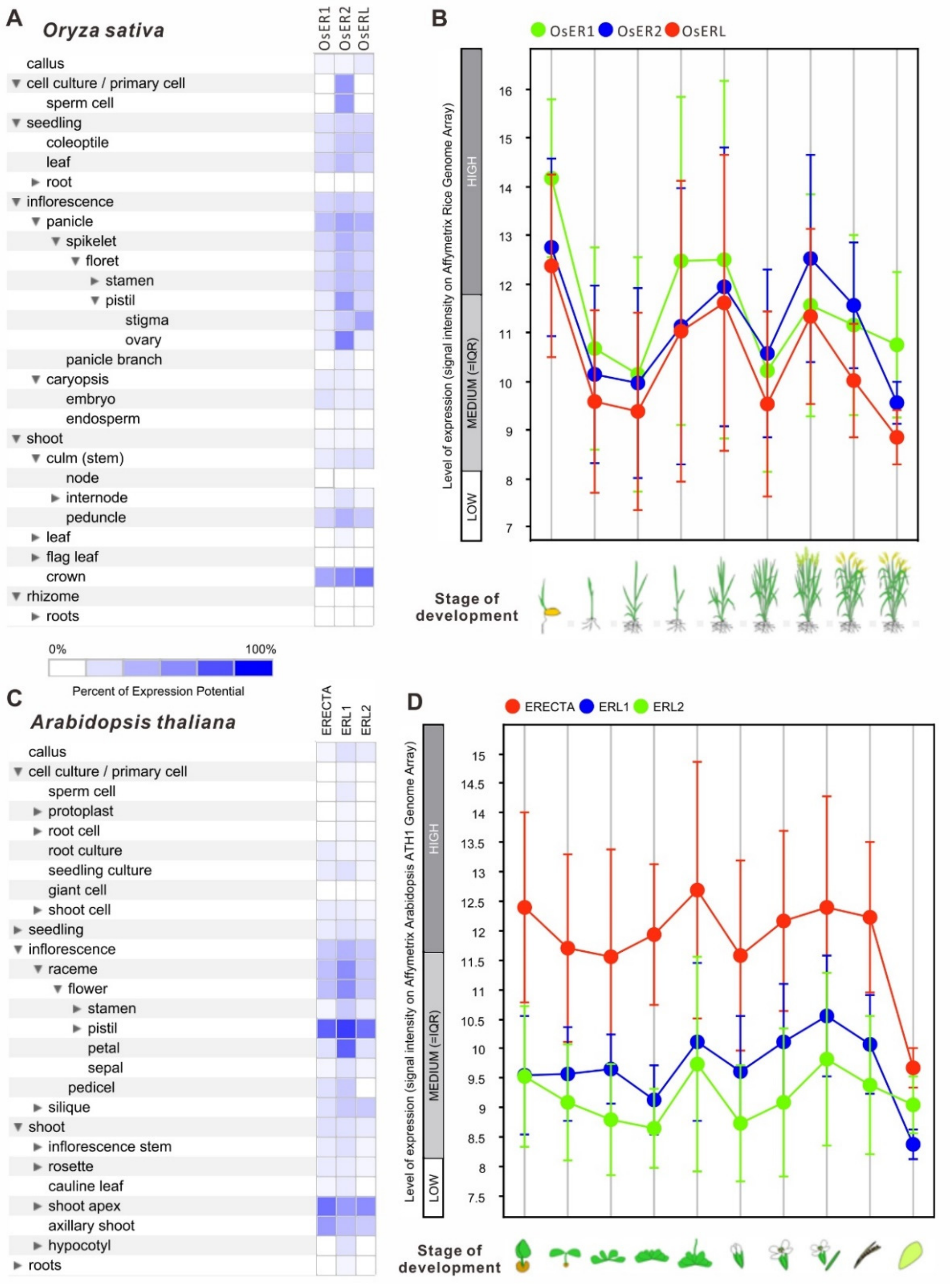

References

Hruz, T., Laule, O., Szabo, G., Wessendorp, F., Bleuler, S., Oertle, L., et al. (2008). Genevestigator V3: A Reference Expression Database for the Meta-Analysis of Transcriptomes. *Adv. Bioinforma.* 2008, e420747. doi:10.1155/2008/420747.
