# Supplementary material for: Phylogenetic and CRISPR/Cas9 Studies in Deciphering the Evolutionary Trajectory and Phenotypic Impacts of Rice ERECTA Genes
Source: Front Plant Sci. 2018 Apr 10;9:473. doi: 10.3389/fpls.2018.00473 (PMC5902711; doi:10.3389/fpls.2018.00473)

**Figure S4. Phenotypic analyses of *oser1-wu1* and *oser2-wu* mutants. (A)** Comparing of plant height between mutants and WT; **(B)** Comparing of panicle size between mutants and WT; **(C)** Comparing of seed setting rate between mutants and WT. The plant height was measured in ripening phase. Values are means ± s.d., ** indicates significance level for each mutant compared to WT at $P < 0.01$ (two-tailed Students' $t$-test). Sample size is $n$ = 7 for plant height, $n$ = 10 for panicle size and seed setting rate.

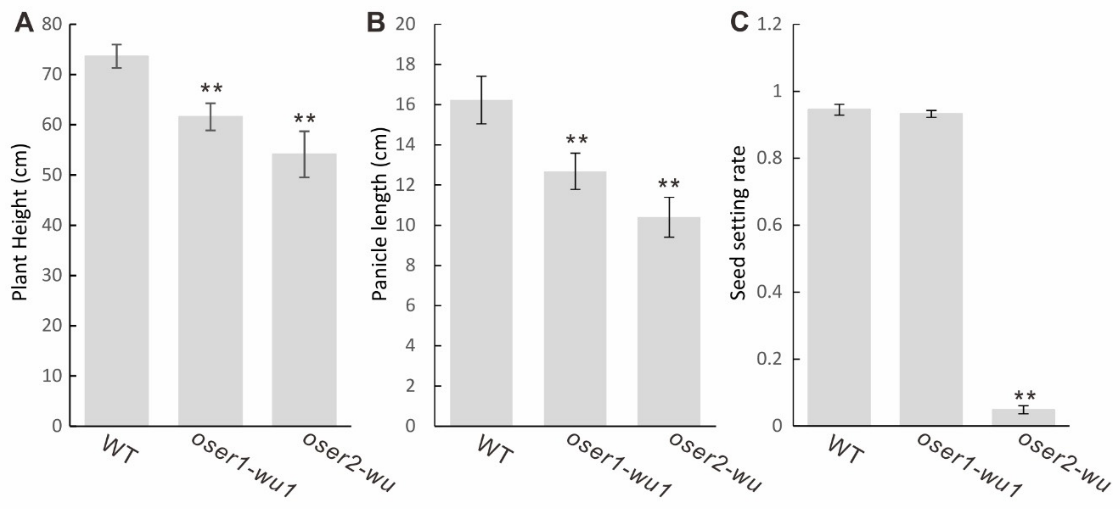

Supplement: Supplementary file 11 [file Image_4.PDF]
