# Supplementary material for: Phylogenetic and CRISPR/Cas9 Studies in Deciphering the Evolutionary Trajectory and Phenotypic Impacts of Rice ERECTA Genes
Source: Front Plant Sci. 2018 Apr 10;9:473. doi: 10.3389/fpls.2018.00473 (PMC5902711; doi:10.3389/fpls.2018.00473)
Supplement: Supplementary file 12 [file Image_5.PDF]

Figure S5. Comparison of different annotation models of *OsER2*. (A) Three gene models of OsER2 given by NCBI (*LOC4330905*), MSU-RGAP release 7 (*LOC_Os02g53720*), and RAP-DB (*Os02g0777400*); **(B)** Spliced junctions of *OsER2* revealed by RNA-seq data. RNA-seq data for Kasalath (young leaves) and Nipponbare (leaf before flowering) were downloaded from NCBI SRA database under accession id DRR013722 and SRR1213582, respectively. The downloaded reads were mapped to Nipponbare reference genome using TopHat version 2.1.0 (Trapnell et al., 2009) with defaults parameters. The mapped results were visualized via the Integrative Genomics Viewer (IGV) (Thorvaldsdóttir et al., 2013). The spliced junctions were in consistence with the NCBI model, suggesting incomplete predictions in both MSU and RAP-DB (with a non-"ATG" start codon) annotations.

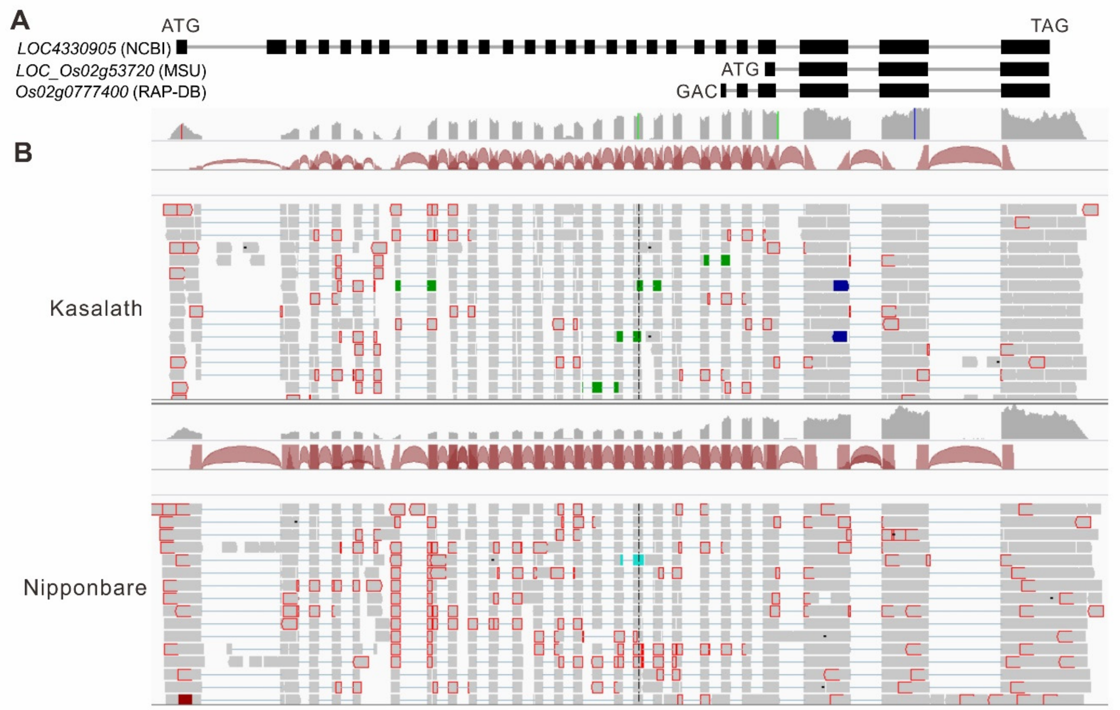

References

Thorvaldsdóttir, H., Robinson, J. T., and Mesirov, J. P. (2013). Integrative Genomics Viewer (IGV): high-performance genomics data visualization and exploration. *Brief. Bioinform.* 14, 178–192. doi:10.1093/bib/bbs017.

Trapnell, C., Pachter, L., and Salzberg, S. L. (2009). TopHat: discovering splice junctions with RNA-Seq. *Bioinformatics* 25, 1105–1111. doi:10.1093/bioinformatics/btp120.
