# Supplementary material for: Phylogenetic and CRISPR/Cas9 Studies in Deciphering the Evolutionary Trajectory and Phenotypic Impacts of Rice ERECTA Genes
Source: Front Plant Sci. 2018 Apr 10;9:473. doi: 10.3389/fpls.2018.00473 (PMC5902711; doi:10.3389/fpls.2018.00473)

Figure S2. Evolutionary characters of *ERfs* within rice population. (A) Phylogenetic tree constructed using consensus protein sequences of 247 rice cultivars for *OsER1*, *OsER2* and *OsERL*. The 247 could be divided into four groups. i.e. *indica* (IND), *aus* (AUS), *temperate japonica* (TEJ) and *tropical japonica* (TRJ), each presents with different colors or shapes. The trees were constructed using the Neighbor-Joining method with 1,000 replicates of bootstrap test. Bootstrap values below 50% are not shown; **(B)** Schematic representation of major polymorphism linkage haplotypes within 247 selected rice cultivars for CDS of three *ERfs*. Only polymorphism loci within CDS are shown for simplification. Each haplotype stands for linked polymorphisms (shown below each exon with rough positions) identified within coding regions. Polymorphism loci which could cause non-synonymous changes are indicated by red asterisks with corresponded protein changes given above.

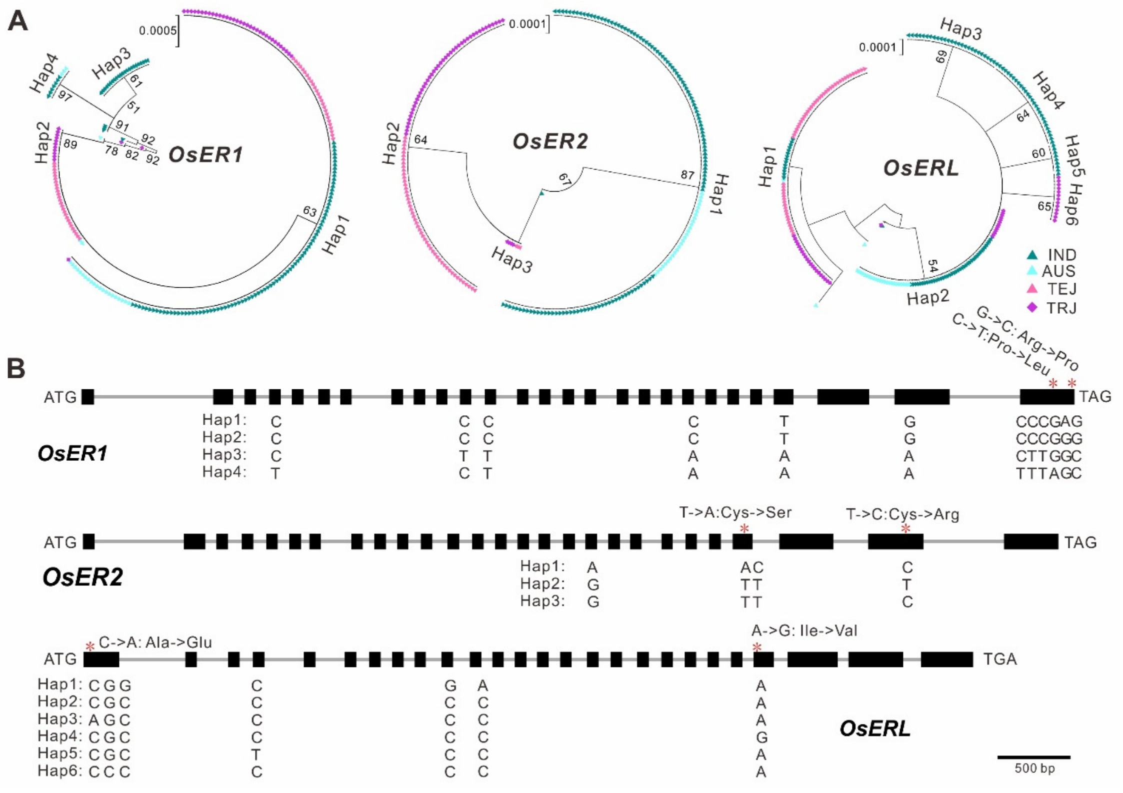

Supplement: Supplementary file 9 [file Image_2.PDF]
